# Green Synthesis of Zinc Oxide Nanoparticles Using Ananas comosus Extract: Preparation, Characterization, and Antimicrobial Efficacy

**DOI:** 10.7759/cureus.47535

**Published:** 2023-10-23

**Authors:** Tanvi Shah, Sugumaran Surendar, Sanyukta Singh

**Affiliations:** 1 Endodontics, Saveetha Dental College and Hospitals, Saveetha Institute of Medical and Technical Sciences, Saveetha University, Chennai, IND

**Keywords:** staphylococcus aureus, antimicrobial potential, ananas comosus extract, zinc oxide nanoparticles, green synthesis

## Abstract

Background

This study aimed to environmentally synthesize zinc oxide nanoparticles (ZnO-NPs) using *Ananas comosus* (AC) extract and evaluated their antimicrobial efficacy against *Staphylococcus aureus*, *Streptococcus mutans*, and *Enterococcus faecalis*.

Methodology

AC extract was combined with a zinc sulfate solution to synthesize ZnO-NPs. The NPs were characterized using UV-visible spectroscopy, Fourier transform infrared (FTIR) analysis, scanning electron microscopy (SEM), and energy-dispersive electron microscopy (EDX). Antimicrobial activity was assessed using the agar disc diffusion method against *S. aureus*, *S. mutans*, and *E. faecalis*.

Results

Green synthesis of ZnO-NPs with AC extract yielded NPs of different sizes and shapes. SEM analysis showed circular and conical NPs measuring up to 10 nm. EDX analysis confirmed the presence of zinc (Zn) and oxygen (O) particles. UV-visible spectroscopy indicated ZnO-NP formation with a peak at 290 nm. These NPs exhibited strong antimicrobial activity against *S. aureus*, with larger inhibition zones at higher concentrations, i.e., 15 mm at 100 μL. Whereas they showed low activity of 12 mm at 100 μL against *S. mutans* and showed no activity against *E. faecalis*.

Conclusions

Environmentally friendly synthesis of ZnO-NPs using AC extract provides an effective method for NP production. It exhibits strong antimicrobial activity against *S. aureus*, indicating the potential for targeted antimicrobial solutions in addressing associated infections.

## Introduction

Nanotechnology is a field that has established itself as a cutting-edge technology with several applications in the chemical, medicinal, mechanical, and food processing industries [1]. Interesting uses of nanotechnology include devices, power production, optics, drug delivery, and ecological studies [2]. The use of nanotechnology to solve a number of urgent issues that individuals face on a daily basis has considerably improved people’s quality of life, such as the demand for more dependable energy sources, the impact of climate change, and advancements in sectors such as beauty, textiles, and healthcare. Over the past decade, there has been a notable emphasis on extensive investigation into metal oxide nanoparticles (NPs) due to their wide range of applications across various technical fields [3]. Zinc oxide nanoparticles (ZnO-NPs) have emerged as an intriguing inorganic material with a wide range of uses, including energy conservation, fabrics, technology, medical care, catalysis, beauty products, semiconductors, and molecular sensing [4]. Conventional methods for the synthesis of NPs include several challenging processes such as the use of hazardous chemicals, prolonged processing times, and high costs which have led scientists to shift their focus toward the development of rapid and ecologically safe technologies for the synthesis of nanomaterials [5]. Due to their unique electronic, optical, and medicinal properties, ZnO-NPs have garnered significant attention in comparison to other NPs [6] as they exhibit high biocompatibility and fast electron transport kinetics, making them suitable for various biological applications, including the construction of biological membranes and other related uses [7].

Green synthesis intends to produce NPs using extracts taken from plants, bacteria, fungi, algae, and other organisms rich in phytochemicals such as polyphenols, terpenoids, flavonoid alkaloids, and sucrose, which function as both reducing and stabilizing agents. Because of these factors, green NP synthesis utilizing plant extracts is developing as a possible trend in green chemistry due to the ease of availability, affordability, and biocompatibility. ZnO-NPs have been manufactured using a variety of plant components, including roots, leaves, stems, seeds, fruits, and peels [8,9]. Bala et al. developed ZnO-NPs by biosynthesis utilizing* Hibiscus sabdariffa* leaves [10]. Aside from being inexpensive, the particles were exceedingly stable and showed potential reactive oxygen species (ROS)-independent antibacterial action. Naseer et al. described the biosynthesis of ZnO-NPs from plant extracts such as *Cassia fistula* and *Melia azadarach*, which were then used as reducing agents [11,12]. The findings demonstrated that plant extracts have a high potential for use as reducing agents in the creation of NPs. While prior research has demonstrated plant-based ZnO-NP production, there is limited literature on their biological capabilities, such as antibacterial, larvicidal, protein kinase, and anticancer properties [13].

*Ananas comosus *(L.) Merr (AC) is the most extensively consumed edible plant in the Bromeliaceae family. It is economically valuable as a widely traded fruit crop and has a long history of usage as a traditional medicine in numerous indigenous cultures [14]. Pineapple crude extract produced from AC contains a diverse range of therapeutic properties. Proteins, minerals, lipids, vitamin C, phenolic compounds, flavonoids, carotenoids, and a number of other nutrients and bioactive chemicals are found in them [15]. AC contains bioactive compounds that have the capacity to decrease metal ions and stabilize NPs during the production process [16]. Pineapple nutrient analysis investigations have shown that such a component can play a critical role as a reducing agent in NP production [17]. Successful ZnO-NP production has been facilitated by green synthesis using pineapple peel extract [18]. Utilizing pineapple fruit extract alongside peel-based synthesis provides a comprehensive approach to ZnO-NP production, ensuring versatility and enabling the tailoring of NP properties to suit a wider array of applications. This dual approach harnesses the full potential of the pineapple plant, minimizing waste and maximizing the utility of its components in nanotechnology [14].

This study aimed to produce ZnO-NPs using AC fruit extract in an environmentally friendly manner, analyze and confirm the properties of the synthesized NPs, and evaluate the effectiveness of the ZnO-NPs against common oral microbes. The goal was to contribute to the development of sustainable NP production and explore potential applications in antimicrobial research.

## Materials and methods

The study was approved by the Review Board of Saveetha Dental College and Hospitals, Chennai (approval number: SRB/SDC/ENDO-2101/22/073).

Preparation of AC fruit extract

**Figure 1:**
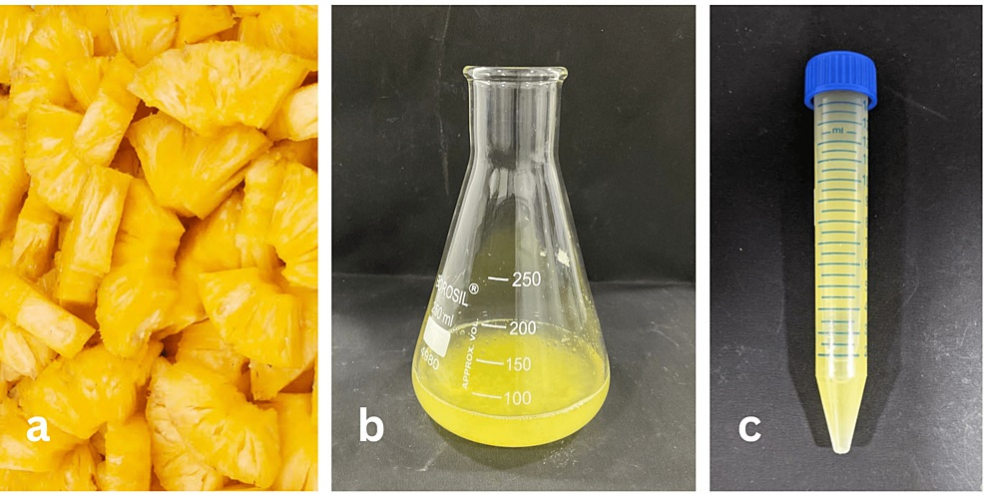
Formation of pineapple extract from pineapple fruit (a), the addition of zinc sulfate solution (b), and the formation of concentrate (c).

The AC fruit was purchased from a nearby fruit shop and cut into clean, fresh pieces. After being crushed, the pulp was passed through a Whatman number 42 filter. To achieve a clear extract, the filtered fruit juice was centrifuged for an additional hour using a hand centrifuge. This extract was stored in the refrigerator (Figure 1).

Preparation of ZnO solution

ZnO-NPs were synthesized using a green synthesis approach mediated by the pineapple extract.

Preparation of Diluted ZnO Solution

For this, 0.57 g of zinc sulfate (20 mmol) was weighed, transferred to a beaker, and diluted to make a volume of 50 mL. The resulting solution was agitated, and the temperature was maintained between 50℃ and 60℃.

Addition of Pineapple Extract to ZnO Solution

The diluted ZnO solution was supplemented with 1 ml of AC fruit extract. In the solution, ZnO-NPs were produced in suspended form. The mixture was stirred gently to ensure uniform mixing of the components. When AC broth was added to an aqueous ZnO solution, the reaction mixture gradually changed color. The NPs were prepared in the experiment, resulting in a clear solution.

Orbital Shaking and Centrifugation

**Figure 2:**
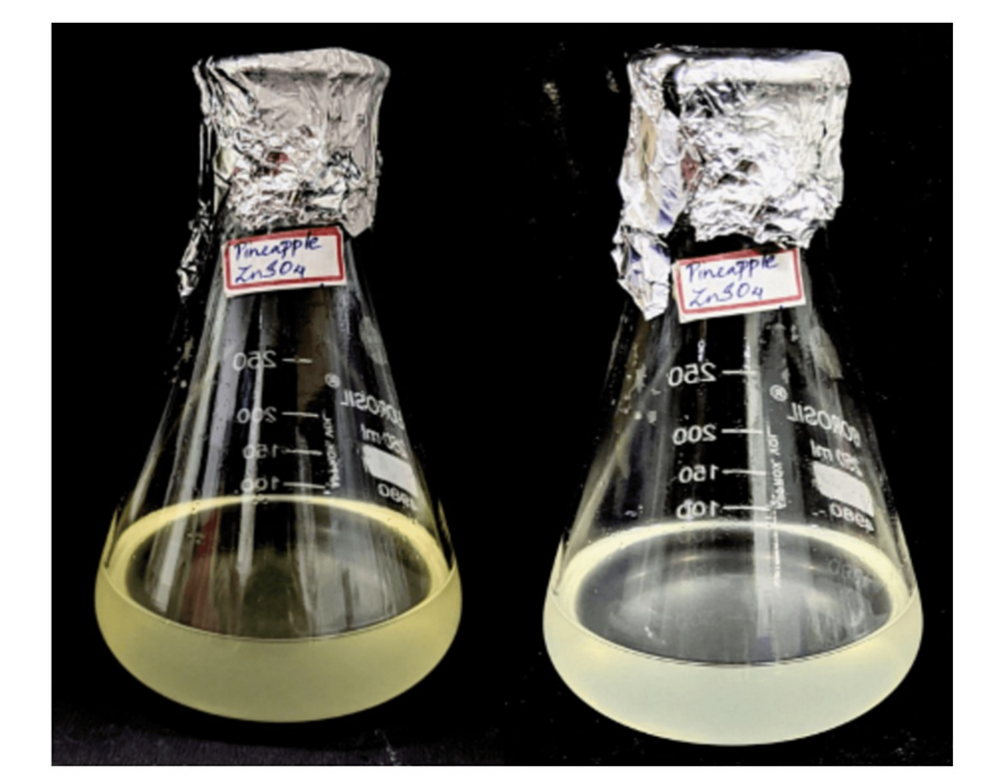
Color changes to a clear solution indicating the formation of ZnO-NPs. ZnO-NPs: zinc oxide nanoparticles

The solution was shaken in an orbital shaker to create homogeneous NPs and then centrifuged at 8,000 rpm for 10 minutes to separate the NPs from the remaining solution. The resulting mix’s supernatant was utilized for characterization, and the residue was a plant extract mediated ZnO-NPs. These were dried at room temperature in the same test tube. One of the earliest indicating signs of the reduction of metal salts into NPs is the visual observation of color change in a solution (Figure 2). This is the endpoint indicator of NP synthesis.

Characterization of NPs

The NPs were subjected to characterization using ultraviolet-visible (UV-Vis) spectroscopy, Fourier transform infrared (FTIR) analysis, and scanning electron microscopy (SEM) analysis, followed by energy-dispersive electron microscopy (EDX). Antimicrobial activity was analyzed by the agar disc diffusion method at different concentrations against common oral bacteria such as *Staphylococcus aureus*, *Streptococcus mutans*, and *Enterococcus faecalis*.

The synthesized ZnO-NPs were subjected to various characterization techniques to evaluate their properties and determine their effectiveness against *S. aureus*, *S. mutans*, and *E. faecalis*.

UV-Vis Spectroscopy

Optical properties and conformation of ZNO-NPs were determined by ultraviolet-visible (UV-Vis) spectrometry (M/S Perkin Elmer, Lambda 25, Waltham, MA, USA) at room temperature. The spectral analysis was performed at the resolution of 1 nm between 200 nm and 900 nm [19].

SEM

SEM (JEOL USA Inc., Peabody, MA, USA) was employed to examine the surface morphology of the synthesized NPs. The synthesized NPs were deposited on a sample holder, coated with a thin layer of conductive material, and analyzed using a high-resolution SEM [20].

Energy Dispersive X-ray Analysis (EDAX)

EDAX analysis was conducted to determine the elemental composition of the synthesized NPs. The NPs were placed on a suitable substrate and subjected to EDAX analysis using an SEM equipped with an EDAX detector (Bruker Germany, D8 Advance Diffractometer, Leipzig, Germany) [21].

Antibacterial Testing

The agar well diffusion technique was used on a Mueller-Hinton agar (MHA) plate to examine the antibacterial efficacy of various dosages of ZnO-NPs against oral pathogens such as *S. aureus*, *S. mutans*, and *E. faecalis*. The MHA was prepared with double-distilled water (pH 7.0) and sterilized in an autoclave at 121°C for 15 minutes. The sterilized MHA was then poured into the petri dish and allowed to harden in a laminar flow at room temperature. A sterile cotton swab wet with microbial culture solution was used to administer an inoculum of 106 cfu/mL of freshly produced bacterial culture to MHA plates. With the assistance of a micropipette, three wells with a diameter of 9 mm were then drilled into the MHA medium and filled with varying volumes (25 μL, 50 μL, and 100 μL) of the final NP solution. The solution was then allowed to diffuse into the medium at ambient temperature for four hours. Subsequently, the culture plates were incubated at 37°C for another 24 hours. After incubation, the diameter of each plate’s zone of inhibition (mm) was measured [19].

FTIR Spectroscopy

FTIR (Thermo Nicolet, Avatar 330, Waltham, MA, USA) analysis was performed to identify the functional groups present in the synthesized NPs. The NPs were mixed with KBr (potassium bromide) and compressed into a pellet for FTIR analysis [22].

## Results

UV-Vis spectroscopy

**Figure 3:**
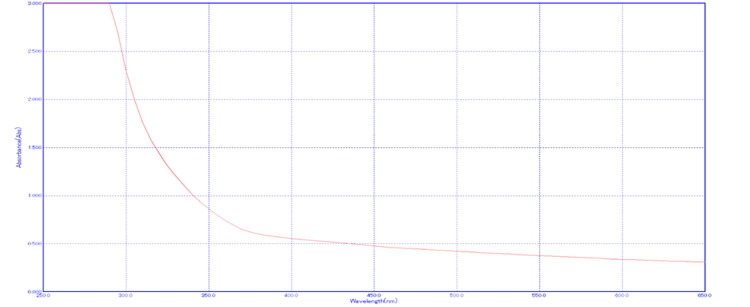
UV-visible spectroscopy showing a single peak at 290 nm wavelength and 3.000 absorbance.

The optical properties of the as-prepared ZnO nanostructure sample were revealed by UV-Vis spectroscopy of green synthesis ZnO-NPs. There was a prominent and conspicuous single peak at 290 nm with an absorbance value of 3.000 (Figure 3). The link between the observed peak and the typical features of ZnO-NPs is a strong indication of their presence and provides persuasive proof for the success of the NP production process.

SEM analysis

**Figure 4:**
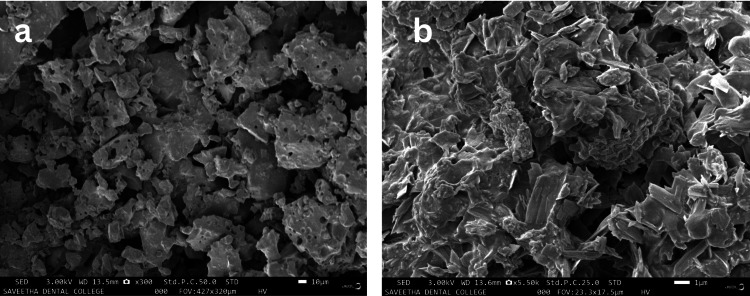
Scanning electron microscopy of samples showing nanoparticles of conical and circular shape at 300× (a) and 5,500× (b) magnification.

The solution was then subjected to drying in a hot air oven, leading to the formation of a white powder. The morphology of the ZnO-NPs was investigated through SEM. They displayed varying sizes and exhibited circular and conical shapes up to 10 nm in size at 5,500× magnification (Figure 4), The average particle size was determined by measuring multiple NPs and calculating the mean.

EDAX

**Figure 5:**
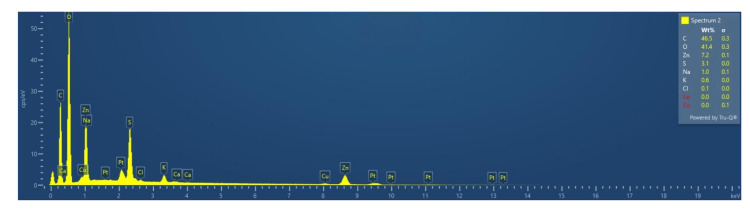
Energy-dispersive electron microscopy results showing quantities of carbon, oxygen, and zinc as 46.5%, 41.4%, and 7.2% (Wt.%), respectively. Wt.%: weight percentage

The EDX peaks were constantly within the ZnO crystallite range. The analysis confirmed the presence of zinc and oxygen, confirming the successful formation of ZnO-NPs. Because the material was inert and pure during the production of ZnO-NPs, no additional peaks were seen in the analysis (Figure 5). This diffraction peak location followed a pattern similar to that seen in the Joint Committee on Powder Diffraction Standards: 36-1451 database (JCPDS).

Agar disc diffusion

The findings indicated strong antimicrobial activity of ZnO-NP solution against *S. aureus* at various concentrations, especially 25 µL, 50 µL, and 100 µL corresponding to a ZOI of 12 mm, 13 mm, and 15 mm, respectively. These measurements were made linearly. Furthermore, when tested at greater doses, the synthesized NPs demonstrated considerable antibacterial efficacy against *S. mutans*. No ZOI was noted against *E. faecalis* as the well diameter was 9 mm, demonstrating a lack of activity against this bacterial strain even at the highest tested doses (Table 1).

**Table 1:** Antimicrobial efficacy of nanoparticles against S. aureus, S. mutans, E. faecalis, and control antimicrobial by the agar disc diffusion method. AB: control antimicrobial (0.2% chlorhexidine) The distance measured is the diameter of the zone of inhibition noted which includes 9 mm well diameter.

Organism	Volume of sample			AB
	25 µL	50 μL	100 μL	
S. mutans	9 mm	9 mm	12 mm	30 mm
S. aureus	12 mm	13 mm	15 mm	41 mm
E. faecalis	9 mm	9 mm	9 mm	35 mm

FTIR spectroscopy

FTIR was performed to identify functional groups in biomolecules responsible for the bioreduction of ZnO and the capping/stabilization of ZnO-NPs. The intense bands observed in measurements were compared with standard values to determine the presence of specific functional groups.

**Figure 6:**
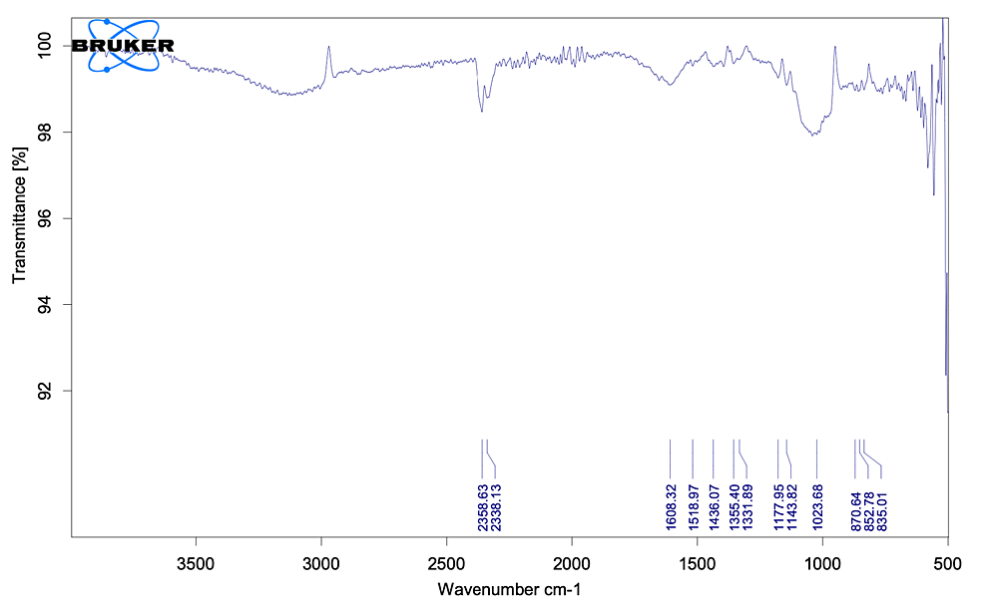
Fourier transform infrared results of pineapple-derived zinc oxide nanoparticles.

The distinctive functional groups linked with the ZnO-NPs were discovered using FTIR studies (Figure 6). The peak at 835 cm^-1^ corresponds to metal-oxygen bond stretching/vibration in ZnO. The peak at 2,358 cm^-1^ relates to carbon residues found during the sample analysis, while the peak at 1,143 cm^-1^ refers to C-O elongation. The stretching vibration of hydroxyl compounds is responsible for the presence of hydrogen bonds at 1,331 cm^-1^ and 1,608 cm^-1^.

## Discussion

Before this study, there has been a lack of research on the green synthesis of ZnO-NPs mediated by fruit extract of AC (pineapple), as older studies made use of its peel extract for the synthesis of ZnO-NP as well as silver NPs [23]. The emergence of antibiotic-resistant strains raised the number of fatalities and the severity of bacterial illnesses dramatically. Patient fatalities from antibiotic-resistant bacterial strains outnumber worldwide deaths from cancer and diabetes mellitus combined [24]. This necessitates the need for the production of safer alternatives that can combat microorganisms involved in oral infections. The FTIR analysis confirmed the presence of functional groups derived from the bioactive compounds in the pineapple extract, which played a crucial role in the reduction and stabilization of ZnO-NPs. The existence of ZnO-NPs with sizes ranging from 10 to 50 nm was confirmed in the investigation. The EDAX analysis showed the presence of zinc and oxide elements and confirmed the successful formation of ZnO-NPs. The spherical shape and uniform size distribution observed in the SEM images indicate the successful synthesis of well-defined NPs, which further paves the way for the future development of these NPs.

The antibacterial activities of ZnO-NPs synthesized by utilizing AC were investigated in this study at a concentration of 25 µL, 50 µL, and 100 µL. The antibacterial activity was assessed using the agar disc diffusion method, with the ZOI diameter quantified in millimeters. At 100 µL, the most significant antibacterial impact was detected against *S. aureus* (15 mm), followed by *S. mutans* (12 mm), while the least effect was recorded against *E. faecalis* (9 mm). The antimicrobial properties of the pineapple extract can be primarily attributed to the presence of the acidic nature of the extract, the phytochemical factors such as flavonoids and vitamin C, and the presence of bromelain, a proteolytic enzyme, which plays a crucial role in breaking down proteins in bacteria, particularly the peptidoglycan cell wall [14]. This interaction with the bacterial cell wall disrupts membrane permeability, leading to significant damage and ultimately cell death [25]. ZnO-NPs have also shown antibacterial action against *Streptococcus pyogenes*, *Escherichia coli*, *S. aureus*, *Klebsiella aerogenes*, *Pseudomonas aeruginosa*, *Proteus mirabilis*, *Mycobacterium tuberculosis*, and *Bacillus subtilis* [20].

Pineapple-derived ZnO-NPs have significant antibacterial capabilities, making them a viable treatment for *S. aureus*-related illnesses. These NPs may be used efficiently in a variety of media for specific purposes. In topical formulations such as ointments and creams, they can inhibit the growth of *S. aureus* on the skin, presenting a potential treatment for cutaneous infections [26]. Incorporating these NPs into oral hygiene products such as toothpaste and mouthwash can help combat *S. aureus* colonization in the oral cavity, preventing oral infections [27]. Furthermore, their integration into wound dressings and medical device coatings holds promise for the prevention and treatment of *S. aureus*-associated infections in healthcare settings [28]. The superior antibacterial activity of pineapple-derived ZnO-NPs offers up new avenues for combating pathogen-related diseases. Green ZnO-NP production with AC extract can be used as an alternative to chemical techniques. ROS generation by ZnO-NPs can result in oxidative stress in the bacterial cell, inhibiting protein synthesis and DNA replication [25].

Limitations of this study involve its in-vitro nature due to which it cannot capture the true essence of the complexities that might arise when these NPs are used in a day-to-day clinical scenario. An extensive study suggested an effect of synthesis temperature on the size and shape of ZnO-NPs which needs to be taken into consideration [29]. There is a need for more tests on antimicrobial activity, not only against common oral microbes but also on different bacterial organisms such as *S. pyogenes*, *K. aerogenes*, and *P. aeruginosa* as well as localized infections of other parts of the body along with systemic infections. Cytotoxicity testing is also necessary for further development of the product [30]. At last, there is a constant requirement for the development of an eco-friendly and commercially viable approach that explores the potential of such natural agents that aid in the synthesis of NPs under exploration.

## Conclusions

The synthesis of AC fruit extract-derived ZnO-NPs is quick and easy, with the fruit extract acting as both reducing and stabilizing agents for the manufacturing of stable NPs. ZnO-NPs have promising antibacterial properties against *S. aureus* and *S. mutans* providing an opportunity for its use in various medical avenues. Further laboratory studies and clinical testing followed by product development are essential to incorporate the ZnO-NPs for patient use specifically in oral hygiene products.
